# Monitoring of the Venezuelan exodus through Facebook’s advertising platform

**DOI:** 10.1371/journal.pone.0229175

**Published:** 2020-02-21

**Authors:** Joao Palotti, Natalia Adler, Alfredo Morales-Guzman, Jeffrey Villaveces, Vedran Sekara, Manuel Garcia Herranz, Musa Al-Asad, Ingmar Weber

**Affiliations:** 1 Qatar Computing Research Institute, HBKU, Doha, Qatar; 2 Massachusetts Institute of Technology, Cambridge, United States of America; 3 UNICEF, New York, United States of America; 4 iMMAP Colombia, Bogotá, Colombia; 5 Global Protection Cluster, Geneva, Switzerland; University of Warwick, UNITED KINGDOM

## Abstract

Venezuela is going through the worst economical, political and social crisis in its modern history. Basic products like food or medicine are scarce and hyperinflation is combined with economic depression. This situation is creating an unprecedented refugee and migrant crisis in the region. Governments and international agencies have not been able to consistently leverage reliable information using traditional methods. Therefore, to organize and deploy any kind of humanitarian response, it is crucial to evaluate new methodologies to measure the number and location of Venezuelan refugees and migrants across Latin America. In this paper, we propose to use Facebook’s advertising platform as an additional data source for monitoring the ongoing crisis. We estimate and validate national and sub-national numbers of refugees and migrants and break-down their socio-economic profiles to further understand the complexity of the phenomenon. Although limitations exist, we believe that the presented methodology can be of value for real-time assessment of refugee and migrant crises world-wide.

## Introduction

The current economic and political crisis in Venezuela has led to an outpouring of refugees and migrants from the country. As of February 2019, the Regional Inter-Agency Coordination Platform for Refugees and Migrants from Venezuela (R4V) estimates that there are 3.4 million people who have left Venezuela and who currently live in countries in Latin America and the Caribbean region [1].

According to the United Nations High Commissioner for Refugees (UNHCR), “this is the largest exodus in the recent history of Latin America” [2]. This has prompted humanitarian interventions from governments, UN agencies and civil society. Targeted efforts, however, have been hampered by missing, outdated or incorrect data concerning the (i) absolute number, (ii) spatial distribution, and (iii) socio-economic composition of Venezuelan refugees and migrants.

Attempts to measure the size of the population displacement have been conducted by governments in the receiving countries. Colombia, for example, ran the *Registro Administrativo de Migrantes Venezolanos* (RAMV) from April to June, 2018, a registry campaign in which Venezuelans living in Colombia could voluntarily identify themselves at specific registration points designated by the government [3]. While this approach provides a snapshot of the crisis, it is limited in many ways. First, it only provides a single snapshot of refugee and migrant stocks and is therefore inadequate to capture sudden flows, e.g., due to an unforeseen deterioration of the situation in Venezuela. Second, the effort of staffing one thousand registration posts for 61 days comes with a significant economic burden. Third, and perhaps most crucially, it relies on self-reported data, which is likely to be incomplete, leading to a considerable under-count of the affected population. One reason for the lack of participation in voluntary registration campaigns such as RAMV is the perceived lack of benefits to registering, in particular for refugees and migrants with an irregular status.

For these reasons, which largely apply to displacement and migration monitoring in general [4], researchers have explored non-traditional data sources for monitoring trans-national mobility [5], including IP addresses of email users [6], geo-tagged tweets [7–11], satellite data to count structures in refugee settlements [4, 12] and Facebook’s advertising audience estimates [13]. In this work, we build on [13, 14] and show how data from Facebook’s advertising platform can be used to supplement the existing monitoring frameworks. Here we focus on the Venezuelan crisis and provide insights into (i) the temporal trends of refugee and migrant flows, (ii) the spatial distribution of Venezuelan nationals in their host countries, and (iii) the socio-economic makeup of these communities. Facebook is a particularly promising data source for this setting as, according to a 2018 survey [15], 70% of the population in Venezuela uses Facebook. Twitter might also be a promising data source, and other researchers are investigating its use for monitoring the Venezuelan exodus [8, 9], though its usage across Latin America (12%) is only one fifth of Facebook’s (60%) [15].

Facebook’s advertising platform is designed to support targeted advertisements based on a large number of user attributes, including self-reported attributes (e.g., age, gender, education, relationship status), inferred attributes (e.g., topical interests, political orientation, cultural affinities) and automatically extracted ones (e.g., device and connection type used to access Facebook). For instance, a person planning to launch an advertisement campaign on Facebook can choose to selectively target people who, according to Facebook’s classification, (i) are aged 13 and above, (ii) are currently living in the Colombian department of Norte de Santander, (iii) used to live in Venezuela, and (iv) primarily use an iOS device to access Facebook. Before the advertisement campaign is launched, Facebook provides the advertiser with an estimate of the number of monthly active users (MAUs) matching the provided targeting criteria. In this concrete example, the estimate is 3,000 MAUs (as of February 24, 2019). Note that 13 is the minimum age set by Facebook for a person to create a profile. These estimates of user counts are available free of charge through the Facebook Graph API [16]. According to the documentation, the numbers we collect are the “estimated number of people that have been active on your selected platforms and satisfy your targeting spec in the past month” [17]. For a more detailed list of possible targeting criteria and how to use the Facebook API please refer to its online documentation [18] and pySocialWatcher documentation [19], the Python wrapper used in this work to collect the data.

## Results

**Fig 1:**
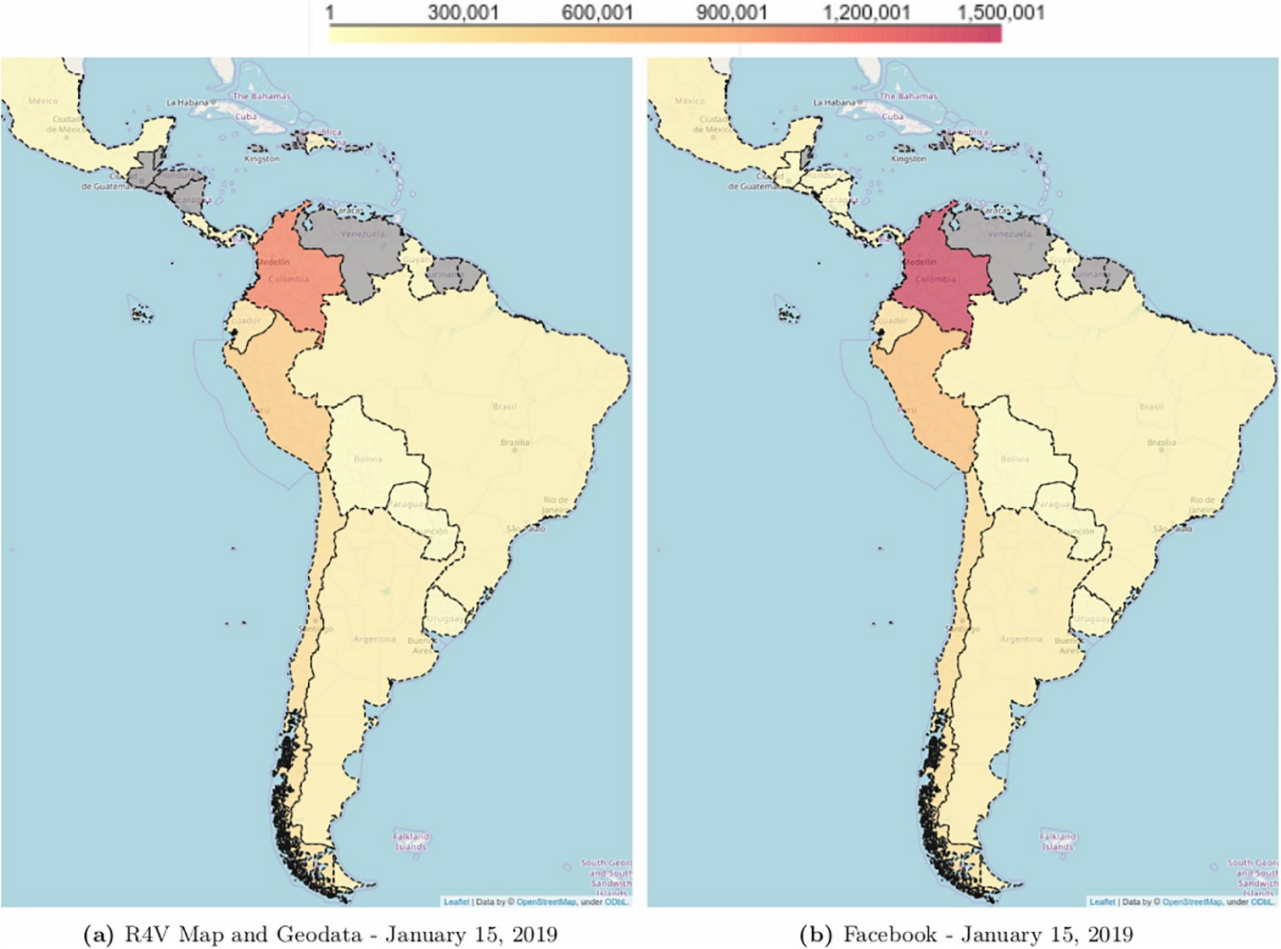
Estimates of Venezuelan refugees and migrants in South America. (A) Estimates from Response for Venezuela (R4V); (B) Estimates from Facebook. Pearson correlation *r* = .99 (n = 17), Kendall correlation of *τ* = .79 (n = 17). Countries are shown in grey when no estimates are available (Fig 1-A) or when Facebook’s returned estimate is smaller than the minimum resolution of 1,000 monthly active users (Fig 1-B).

**Fig 2:**
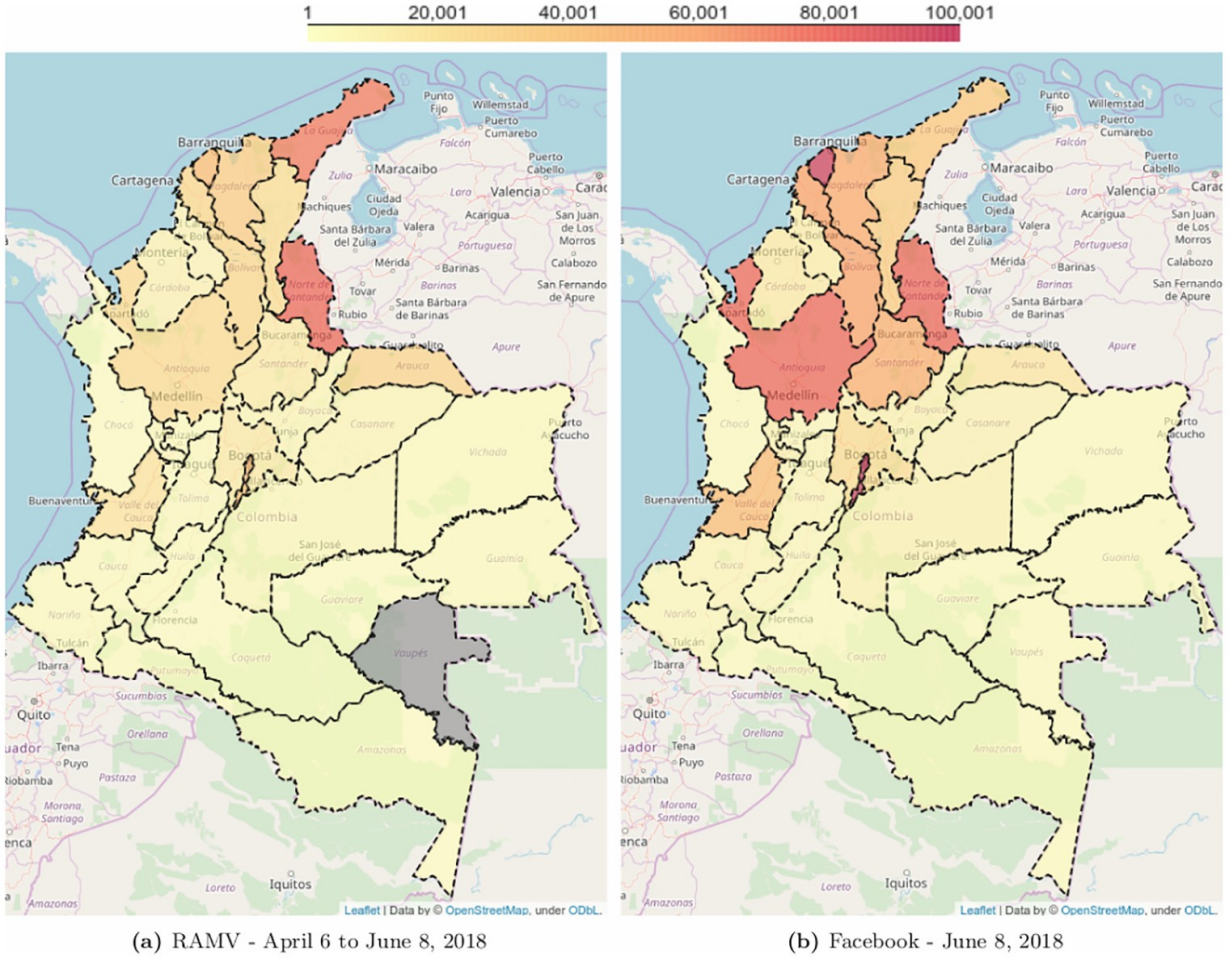
Estimates of Venezuelan refugees and migrants in Colombia. (A) Estimates from the Registro Administrativo de Migrantes Venezolanos (RAMV); (B) Estimates from Facebook. Pearson correlation of *r* = .57 (n = 31), Kendall correlation of *τ* = .71 (n = 31).

To understand if, despite its limitation to Facebook users and not the general population, these estimates of user counts are capturing the magnitude of the actual migration, Figs 1 and 2 show a comparison for the general “Facebook users aged 13 and above who used to live in Venezuela” with the most recent official estimates.

Fig 1 shows the estimates of Venezuelans living in Latin American countries according to the latest report from the Regional Inter-Agency Coordination Platform for Refugees and Migrants from Venezuela (R4V) [20] (Fig 1A) and Facebook Advertisement (Fig 1B), both from the same period, January 15, 2019. The two data sources share a similar spatial distribution with a Pearson and Kendall correlation across the different countries of, respectively, *r* = 0.99 and *τ* = 0.79 (n = 17, p < .0001 for both metrics). In January 2019, R4V Map and Geodata reported a total of 2.7M Venezuelan nationals across 17 countries [20], while there were an estimated 3.2M Facebook users who previously lived in Venezuela now living in of these countries.

The advantage of the advertising API is that it allows the disaggregation of numbers to sub-national levels. At the sub-national level, Fig 2 compares the estimates of Venezuelan nationals (left, according to RAMV) and Facebook users who used to live in Venezuela (right, according to Facebook). The Pearson correlation of *r* = .57 (n = 31, p = .0008) between RAMV and Facebook is smaller than for the continental data above. The decrease in the Pearson correlation coefficient can be noted in Fig 2, in which Facebook estimates more refugees and migrants from Venezuela than RAMV for several departments away from the Venezuela-Colombia border. The largest absolute difference was found for capital district of Bogotá (Distrito Especial, Colombia), where Facebook estimated 490k refugees and migrants from Venezuela, while RAMV reported only 43k. Part of this difference might be explained by the number of registration stations to conduct RAMV: 223 for the department of Norte de Santander, where RAMV reported 82k Venezuelan nationals, and only 59 for the Distrito Especial. We also highlight that a Kendall’s rank correlation of *τ* = .71 (n = 31, p < .0001) shows that the agreement for the relative rank among the departments of both distributions is high. Thus, even if the estimates from RAMV were perfect, Facebook-derived estimates would still be a useful tool to discover where the density of migrants is higher, allowing a better targeted management of the refugee and migration crisis.

**Fig 3:**
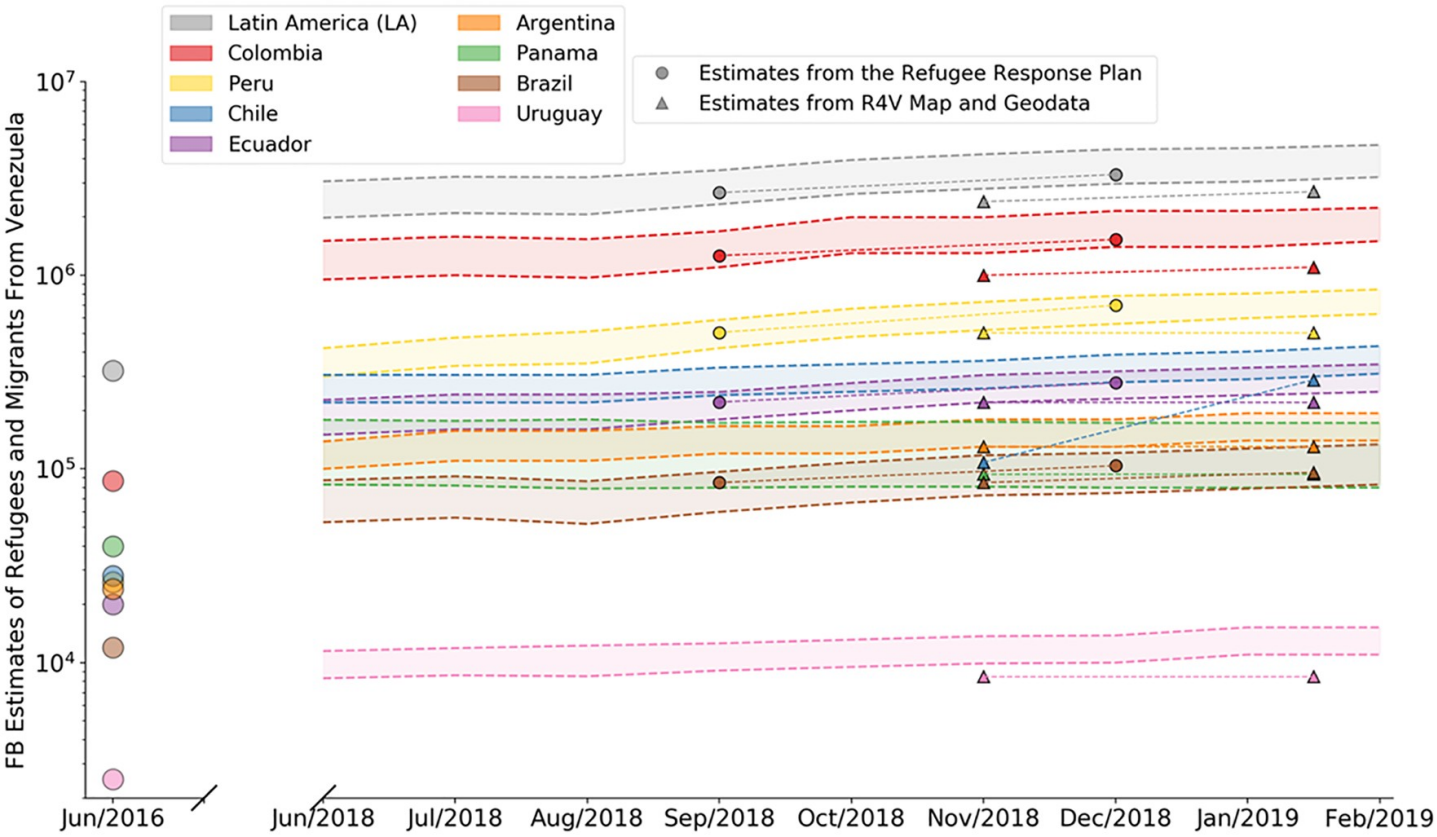
Migration trends for Venezuelans in Latin America. Lower estimates are the raw estimate from the Facebook Marketing API, whereas upper estimates take into consideration a correction factor for Facebook penetration in the host countries. Estimates for refugees and migrants from Venezuelans in Latin America compiled in the R4V Map and Geodata [20, 22] and the Refugee Response Plan [21] are shown for comparison.

Concerning the temporal evolution of the Venezuelan refugee and migration crisis, Facebook’s advertising platform is more limited as it does not offer any historical information. For example, one cannot obtain estimates on the number of Facebook users who previously lived in Venezuela and who lived in Cúcuta, Colombia, in August 2014. Temporal trends can, however, be traced through repeated data collections, eventually building up a historic repository. Fig 3 shows Facebook-derived estimates for eight countries in Latin America compared to estimates by R4V. Note that even though both the “Response Plan” [21] and the “Map and Geodata” [20, 22] are released by R4V, their numbers are not fully consistent. The lower bounds for the bands in Fig 3 are the raw population estimates from Facebook, i.e., estimates for the number of monthly active Facebook users aged 13 and above who used to live in Venezuela but who now live in one of the host countries. The upper bounds represent estimates corrected for Facebook penetration, assuming that Venezuelan refugees and migrants are as likely to join Facebook as the population in the host country. See Methods and materials for details on how the Facebook penetration in the host countries is taken into account. The estimates from June 2016 were originally collected for [13] and the data was shared by the authors.

In [13] (Fig 2) the authors observed that raw estimates derived from the Facebook Marketing API for the percentage of the population that is foreign-born are *overestimates* when compared to migration data from the World Bank. This holds in particular in Latin American and African countries, but less so in Europe and North Africa. Put differently, prior research suggests that in Latin America migrants are more likely to use Facebook than non-migrants. If also true for Venezuelan refugees and migrants, using the Facebook penetration of the total resident population as a correction factor will yield upper bounds and the true numbers will fall inside the bands of Fig 3.

Based on the spatial (Fig 1) and temporal (Fig 3) comparison to the best available data from R4V Map and Geodata, the Response Plan and RAMV, the estimates from Facebook are a useful proxy for the number of Venezuelan refugees and migrants. Note that the lack of reliable “gold standard” data to use for validation is the main motivation to consider non-traditional data sources to triangulate existing ones.

Whereas the focus above was on validating, where possible, estimates obtained from Facebook, the following analyses focus on obtaining estimates for aspects where no comparable data exists. These include (i) sub-national estimates for the spatial distribution and (ii) insights into the socio-economic status of Venezuelan refugees and migrants in different host countries.

**Fig 4:**
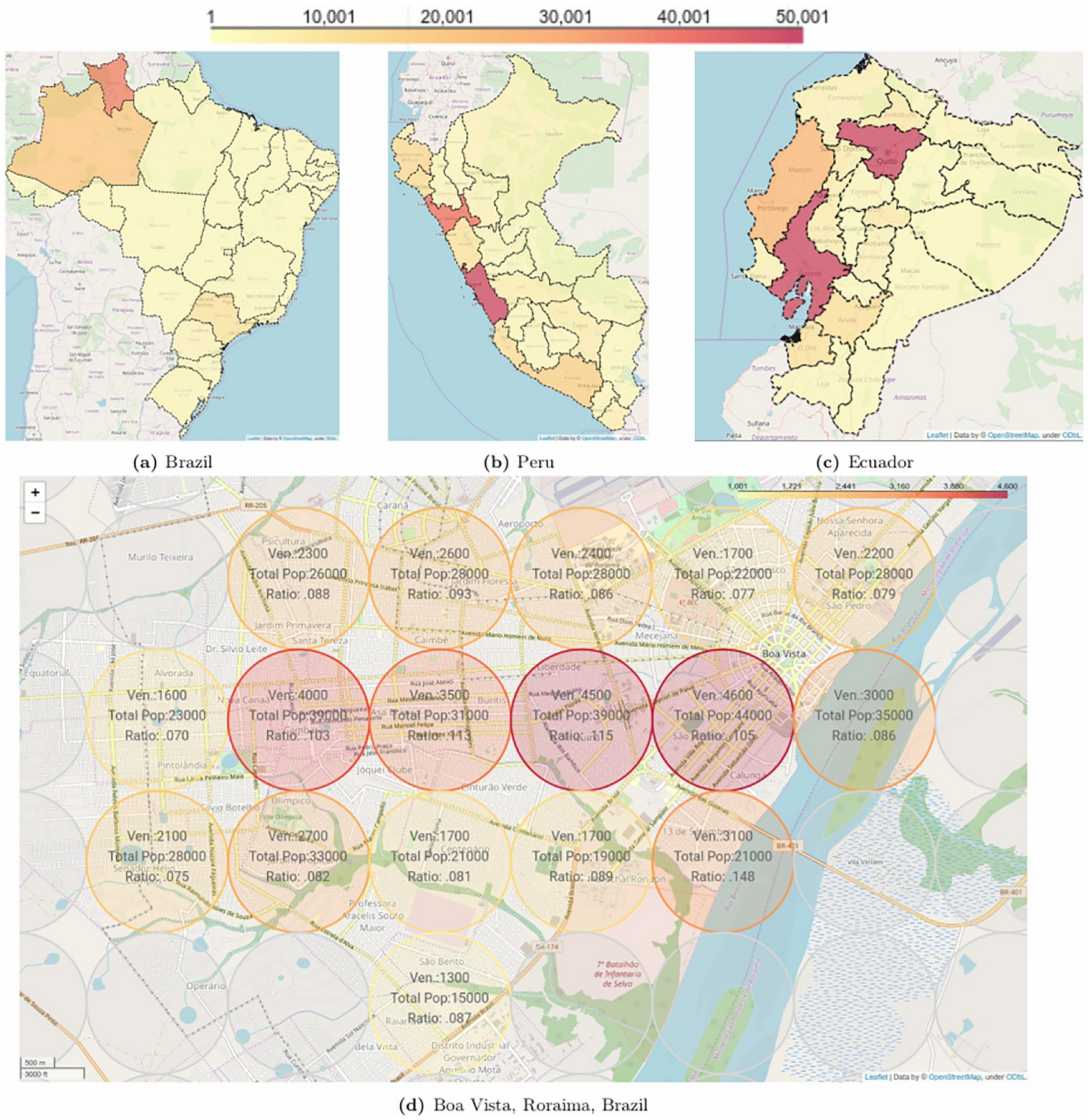
Raw estimates of Venezuelan refugees and migrants in regions where no other data is available. Top: Brazil, Peru and Ecuador; Bottom: Boa Vista, Brazil. Data collection was done through Facebook Marketing API on February 18, 2019.

Fig 4 shows estimates, obtained from Facebook, for the spatial distribution of Venezuelan refugees and migrants at the highest sub-national administrative level, i.e., across provinces (“provincias” in Peru and Ecuador) or states (“estados” in Brazil). For example, the top left map of Fig 4 shows that, based on Facebook-derived estimates, around 75% of the refugees migrants from Venezuela in Brazil are in two states on the Brazil-Venezuela border, Roraima and Amazonas. Further in the South of Brazil, the richer states of Sao Paulo and Rio de Janeiro are home to 12% and 5% respectively of the Facebook users who previously lived in Venezuela. Likewise, the top middle and top right maps of Fig 4 provide a state-level analysis of the estimated spatial distribution of Venezuelan refugees and migrants across Peru and Ecuador. Finally, to illustrate the spatial resolution that can be obtained via Facebook’s marketing API, the bottom map of Fig 4 shows a breakdown of the Brazilian city of Boa Vista in the state of Roraima, where most of the migrants from Venezuela are currently located. The maximum resolution allowed by Facebook is a 1-km radius circle centered on a given latitude and longitude coordinate.

**Fig 5:**
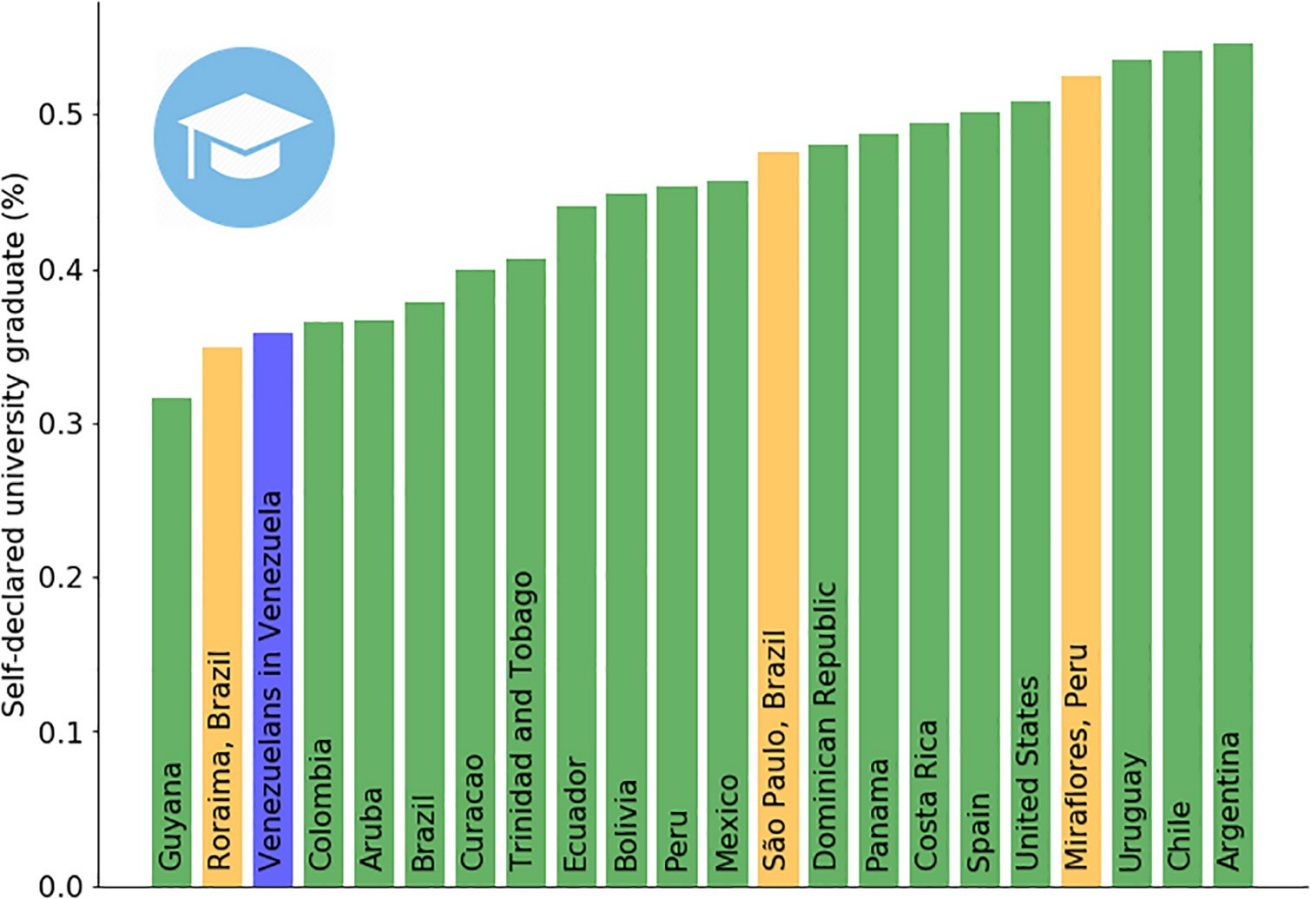
Percentage of university graduate refugees and migrants from Venezuela in different countries. Data collected through Facebook Marketing API on Feb. 18, 2019.

**Fig 6:**
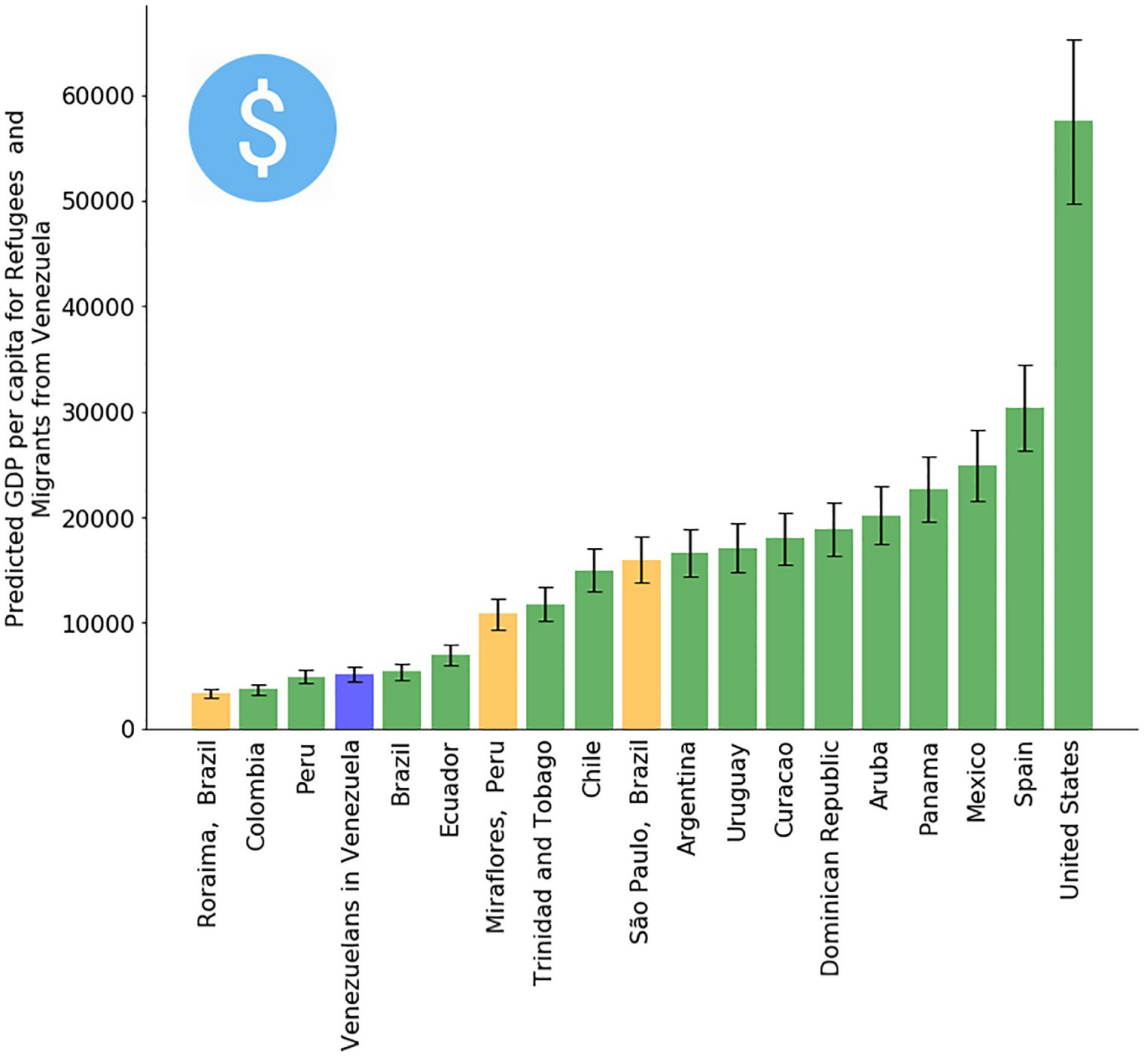
Estimated nominal GDP per capita for refugees and migrants from Venezuela. The linear model created to generate these estimates is based on the percentage of iOS devices for the host population in each country. Errors bars of 13% were calculated using the symmetric mean absolute percentage error from the training set. Details in Materials and Methods and S1 File.

Estimating the absolute number and the spatial distribution of Venezuelan refugees and migrants are a top priority in order to quantify the magnitude of the crisis and to plan an appropriate humanitarian response. However, insights into their socio-economic status and how it compares to the host population are also important, in particular to anticipate potentially hostile sentiments from the host population [23, 24]. To illustrate how Facebook’s audience estimates can be used for this purpose, Figs 5 and 6 provide an exploratory socio-economic analysis of the Venezuelan population in Latin American countries, analyzing their self-reported education level (Fig 5) and their inferred average income per capita (Fig 6)—details in the Materials and Methods section below. While this information is hard to validate due to the lack of official data, there are sources that the results can be contrasted with. For example, historically countries like Panama and Costa Rica have received wealthier and educated Venezuelans [25], while countries like Colombia or Peru are recently receiving poorer, less educated ones [26, 27]. In Chile, the number of professionals and educated refugees and migrants that seem to be underemployed is consistent with recent UN Venezuelan migrant reports [28]. Concerning the inferred average income per capita, we note that the *absolute* dollar values are likely at best indicative, rather than accurate. However, we observed the *relative* grouping of countries to be stable. Concretely, irrespective of whether using (i) connectivity type, (ii) mobile operating system, or (iii) a grouping of devices by price to build models, the following three groups of host countries always maintained their relative order to each other, though not necessarily within each group. Bottom group: Brazil, Colombia, Ecuador, Peru. Middle group: Argentina, Chile, Costa Rica, Mexico, Panama. Top group: Spain, United States. Based on the model stability of these three strata, we believe that they are likely to reflect real differences in average socio-economic situation.

## Discussion

In this work, we showed the benefits of triangulating emerging sources of data, such as Facebook’s advertising data, to supplement official refugee and migration statistics. In the context of the Venezuelan crisis, Facebook’s advertising data has proved valuable because of its low latency (days not months), low acquisition cost (only programming efforts), high spatial resolution (sub-city resolution), and possibility to disaggregate by socio-economic status (education level and inferred income), as well as the remote sensing capabilities (reduce the need to visit unsafe regions). The global reach of Facebook operations also provides an advantage over other types of data aggregation, such as mobile phone data (e.g., call detail records (CDR)) and census data. While CDR have been used to successfully map population distribution and mobility *within* a country [29–33], they are typically limited to a single country as aggregating CDR data across countries comes with both technical and legal difficulties. This limits their use to study *cross-border* displacements [34].

In fact, even “gold standard” cross-country migration data in official statistics is subject to inconsistency in availability, definition, and quality [35–37]. An example of this problem is shown in Fig 3, in which the R4V and Refugee Response Plan estimates differ. One reason is that both rely on aggregated counts from different sources, such as statistical or migration departments or federal police. These entities in turn rely on different counting or estimation methods, ranging from voluntary self-registrations to census enumerations, to sample-based surveys and port-of-entry tracking. Census data is also of limited value in understanding fast-evolving migration crises due to the slow update cycle. Case in point, the most recent data on migrant stocks collected through national censuses and available through the United Nations (http://data.un.org/) [38] date from 2007 to 2015 for Argentina, Brazil, Bolivia, Chile, and Peru, long before the apex of the Venezuelan crisis. Note that though Colombia performed its national census in 2018 (https://www.dane.gov.co/), this data has not yet been made available through http://data.un.org/. Facebook data, though based on a black box, offers the convenience of being timely and based on the same estimation methodology, which is a desirable feature for migration analysis [39].

A concrete example of the operational benefits of our methodology is the understanding of the spatial distribution of Venezuelan refugees in Brazil (see Fig 4). Knowing this distribution helped redefine and amplify the geographical scope of humanitarian and longer-term development interventions beyond the border-crossing area. The recognition of the national scale of the crisis was particularly relevant for UNICEF to develop anti-xenophobia campaigns using Facebook’s chatbots.

Despite the advantages outlined above, it is important to acknowledge limitations of using non-traditional data sources [40]. One key limitation is the dependence on Facebook’s inaccessible algorithm for identifying users’ previous countries of residence. Although hints of this procedure can be obtained from academic work published by Facebook researchers [41], indicating that both the self-declared “home” location and social network structure play a role, the exact set of features or the evaluation used for the inference remains hidden. Regarding privacy concerns, the risks are relatively small as only anonymous and aggregate data is obtained—namely the number but not the identities of Facebook users matching provided targeting criteria. Previous researchers who identified privacy leaks in Facebook’s advertising platform [42] had used so-called “custom audiences”, built around mobile phone numbers or email addresses of known users, which is not done for this line of work.

Another technical limitation is the lack of availability of historic data through the advertising platform, meaning that temporal trends can only be inferred from building up a repository over time. Monitoring long-term temporal trends of any platform gets further complicated by typical changes of market shares (MySpace and its decline are a cautionary tale), changes on the usage patterns over time, potentially in response to evolving privacy concerns, and changes on the underlying categorization algorithm of Facebook, requiring re-validating and re-calibrating models built on top of them.

Case in point, shortly after finishing our data collection, Facebook changed their algorithm for labeling users as “lived in Venezuela” around March 15, 2019. This led to a sudden drop from 3.2M Venezuelans living in Latin America on March 14th to 2.4M on March 17th. During this roll-out the spatial distribution remained virtually unchanged though with Pearson correlation of *r* = .99 across 17 countries. However, during our period of analysis, which ended on February 18, 2019, the absolute relative percentage changes, averaged across the different countries, never exceeded 9%. See Fig B in S1 File for details.

The use—and potential removal—of fake accounts and bots is another potential point for concern. According to Facebook’s own estimates [43] about 5% of monthly active users are caused by such accounts. In Q4 2018 and Q1 2019 Facebook disabled a staggering 3.4 billion accounts suspected of being created automatically, the vast majority shortly after their creation. In this work we have not seen any evidence of bot accounts skewing the estimates. This might, however, change if certain financial or political incentives were tied to the estimates derived from the Facebook Marketing API.

Lastly, there is a risk when using digital traces to monitor humanitarian crises to exclude affected people without access to digital technology—potentially the most disadvantaged—who will not leave traces and hence remain uncounted. In our validation we do however observe that despite the selection bias and potential noise of the algorithm to infer users’ previous countries of residence, the estimates derived from Facebook are close to the best official estimates. This is in line with the observation by Wesolowski et al. that despite the substantial bias in phone ownership, mobility patterns derived from mobile phone data are surprisingly robust [44].

**Fig 7:**
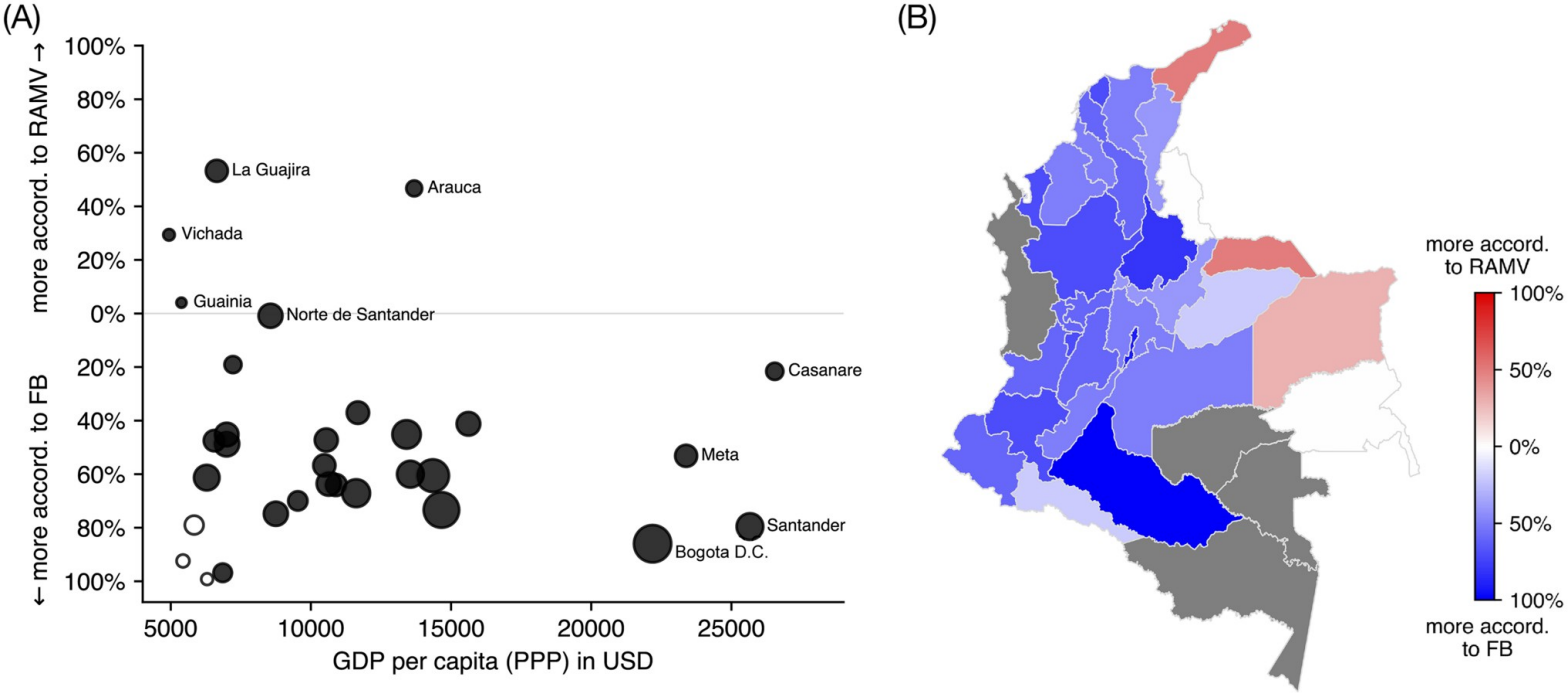
Bias in estimations of migrant and refugee numbers, comparing estimates for Colombia from Facebok and the RAMV survey. (A) Over- and underestimations of populations for individual departments in percentages. If Facebook estimates more refugees and migrants to be in one department we calculate the over-estimation compared to RAMV in percentage. If RAMV estimates more, we calculate the percentage compared to Facebook’s estimate. The size of each point is scaled according to the population of the department. Departments with low numbers of refugees and migrants according to both Facebook and RAMV are colored white (1000 or below). (B) Departments adjacent to the Venezuelan border contain larger number of refugees and migrants than what is reported by Facebook. Departments for which both Facebook and RAMV have little data for are colored gray (1000 or below).

Despite the general good agreement, looking at the differences between estimates derived from Facebook and official estimates reveals important patterns of bias. In Fig 7A we illustrate how the GDP per capita of regions relates to over- and under-estimation bias. We specifically focus on Colombia because of the recent RAMV survey, which currently is the best existing ground truth estimate. Whereas Facebook in general overestimates the number of refugees and migrants, it *underestimates* for certain of the less wealthy departments, such as La Guajira and Vichada. This might, in part, be caused by the geographic proximity of these departments to the Venezuelan border (see Fig 7B), where RAMV efforts might have been more exhaustive. But there is also good reason to assume that the socio-economic situation of the border region contributed to the discrepancy between the two data sources.

At a high level our work demonstrates the value that data held by private companies can have when used for public good [45], in particular in the domain of rapid disaster assessment [46]. We hope that our research helps to further the discussions on how to form Data Collaboratives [47] in support of humanitarian and development projects.

## Materials and methods

### Estimating the number of refugees and migrants from Venezuela

The dataset used in this paper was collected through the Facebook Marketing API (version 3.1) [18]. In this work, we automatically collected data using the Python library pySocialWatcher [19]. This library provides a wrapper for the relevant calls in Facebook’s Graph API [16].

For the purpose of the work presented here, we capture the number of Facebook monthly active users (MAUs) aged 13 or above classified as belonging to the category *Lived in Venezuela (formerly Expats—Venezuela)* for different education levels and access devices across 17 countries (including sub-level country data for selected countries) between May 23, 2018 and February 18, 2019. The category *Lived in Venezuela (formerly Expats—Venezuela)* was renamed *Expats—Venezuela* in late 2018, but its definition remained the same *“People who used to live in Venezuela who now live abroad”*.

We are aware that, technically, the description of the Facebook category used in this work does not perfectly align with the UN definition of either migrant or refugee [48, 49]. According to the UN international recommendations on international migration statistics, a ‘migrant’ is someone who has changed their country of usual residence for more than a year and a ‘refugee’ is someone who has a refugee status granted either before arrival or upon arrival in the receiving country. Nevertheless, our validation results shown in this article encourages us to refer to the *Lived in Venezuela (formerly Expats—Venezuela)* Facebook category as Venezuelans refugees and migrants.

### Correction factor for Facebook penetration in the host countries

When analyzing the temporal trends in Fig 3, we included up-adjusted Facebook audience estimates that correct for the fact that not all refugees and migrants are on Facebook by taking into account the Facebook penetration in the host country. For that, we used the 2017 population estimations compiled by the United Nations [50]. An adjustment factor is calculated for each country as shown in Eq 1. Estimates for refugees and migrants from Venezuela are then corrected as shown in Eq 2.

$$\begin{array}{c} adjFactor(country)=FBAud(country)/UN2017(country) \end{array}$$ (1)

$$\begin{array}{c} venAdj(country)=ven(country)/adjFactor(country) \end{array}$$ (2)

### Facebook data compared to other reports

Table A in S1 File shows the raw Facebook estimates of refugees and migrants from Venezuela as well as the estimates collected by both the Refugee Response Plan (RRP) and the R4V Map and Geodata (R4V) initiatives. The data in this table is the same used in Fig 1. The largest absolute error between the estimates from Facebook and other sources (R4V or RRP) was found in February 2019, when Facebook estimated 1.5M refugees and migrants from Venezuelans in Colombia while R4V estimated 1.1M. The smallest absolute difference occurred in Nov 2019 when both Facebook and R4V estimates coincided in Argentina, 130K. The largest relative error was found in Nov, 2018 in Chile, when Facebook estimated 260k refugees and migrants from Venezuela, 2.4 times more than the R4V estimated of 108K for the same location.

### University graduate users in Facebook

The current Facebook Marketing API version has thirteen non-overlapping categories for education level. Details about the education levels supported by Facebook’s Marketing API can be found online [51]. In this work, the definition of “university graduate” combines the following five categories from Facebook: (1) *“At university (postgraduate)”*, (2) *“Doctorate degree”*, (3) *“Master’s degree”*, (4) *“Some university (postgraduate)”*, (5) *“University graduate”*. Table B in S1 File details information regarding university graduate Facebook users in different locations, both for the host population as well as for the refugees and migrants from Venezuela living in the same location. Part of the data shown in Table B in S1 File can be found in Fig 5.

### Linear regression model to predict GDP

The prediction of the Gross Domestic Product (GDP) per capita at nominal values was conducted with an ordinary least squares linear regression model. We employed as ground-truth data to train the linear regression model the most recent GDP per capita data collection made in 2017 from the United Nations [52]. The linear regression model has only one independent variable *X*, representing the *percentage* of iOS-device users in the considered population. In detail, the targeting attribute used is called *“Facebook access (mobile): Apple (iOS) devices”* and is described as *“People who primarily access Facebook using an Apple (iOS) mobile device”*. The fitted model is *Y* = 507.13+ *X* ⋅ 104903.24. The model, detailed in Table C in S1 File, reached a Mean Absolute Error of 3,782, a Root Mean Squared Error of 4,537 and a Symmetric Mean Absolute Percentage Error (SMAPE) of 13.46%. The SMAPE formula used in this work is $\text{SMAPE}=\frac{100}{n}\sum_{t=1}^{n}\frac{|F_{t}-A_{t}|}{|A_{t}\left|+\right|F_{t}|}$, where *A*_*t*_ is the actual value and *F*_*t*_ is the predictied value. Results range from 0 to 100. Table D in S1 File shows the raw data used in this experiment.

The model above, which predicts a country’s GDP per capita at nominal values, is then applied to the sub-population of Facebook users who used to live in Venezuela and who now live in the different host countries and regions. The predictions for this sub-population can be found in Fig 6.

Apart from the model using only the number of iOS users, we experimented with models with other variables as well. Facebook provides aggregated numbers regarding the type of connectivity (Wifi, 3G, 4G) and for the specific mobile device (e.g., iPhone 6, Samsung Galaxy S7, Sony Xperia Z). We mapped each device model to a price range category according to its market value in December 2018, as shown in Table E in S1 File. We devised 4 different linear regression models with these variables: M-Connectivity model with 3 variables representing the distribution of each connectivity type (3G, 4G, Wifi); M-Price model with 4 variables representing the distribution of price-range category (Expensive, Mid-range, Cheap, Other); M-OperatingSystems with 3 variables representing the distribution of operating systems (iOS, Android, Other); M-All with all previous 10 variables. Fig A in S1 File shows that Kendall’s *τ* correlation for the GDP predictions made by each model compared to each other is high (minimal correlation is higher than .56).

Given that the results of the different models strongly correlate, we opted to show in Fig 6 the results for the simplest model with only the percentage of iOS devices. Apart from avoiding potential overfitting, this simple model is less prone to have resolution problems (e.g., the number of Venezuelans with expensive devices in Roraima, Brazil is smaller than the minimum Facebook resolution of 1000 MAUs).

## Supporting information

S1 File Includes Figs A, B, C and Tables A, B, C, D and E. (PDF)
