# Correction: Monitoring of the Venezuelan exodus through Facebook’s advertising platform

**DOI:** 10.1371/journal.pone.0230455

**Published:** 2020-03-10

**Authors:** Joao Palotti, Natalia Adler, Alfredo Morales-Guzman, Jeffrey Villaveces, Vedran Sekara, Manuel Garcia Herranz, Musa Al-Asad, Ingmar Weber

In the Author Contributions, Manuel Garcia Herranz (MGH) should be listed as having the following contributions: Conceptualization, Formal Analysis, Investigation, Methodology, Validation, Writing–Original Draft, Writing–Review & Editing.
