# Supplementary material for: Monitoring of the Venezuelan exodus through Facebook’s advertising platform
Source: PLoS One. 2020 Feb 21;15(2):e0229175. doi: 10.1371/journal.pone.0229175 (PMC7034953; doi:10.1371/journal.pone.0229175)
Supplement: S1 File — (PDF) [file pone.0229175.s001.pdf]

Supplementary Material: Monitoring of the Venezuelan exodus through Facebook's advertising platform

Joao Palotti[1,2], Natalia Adler[3], Alfredo Morales-Guzman[2], Jeffrey Villaveces[4], Vedran Sekara[3], Manuel Garcia Herranz[3], Musa Al-Asad[5], Ingmar Weber[1*]

1 Qatar Computing Research Institute, HBKU, Doha, Qatar
2 Massachusetts Institute of Technology, Cambridge, United States
3 UNICEF, New York, United States
4 iMMAP Colombia, Bogotá, Colombia
5 Global Protection Cluster, Geneva, Switzerland

* Corresponding author: iweber@hbku.edu.qa (IW)

Assessing the collection variability over time

Snapshots of this migration crisis, as shown in Figs 1-4, are useful for stakeholders as they provide an overview of the crisis in near real-time with high spatial resolution. Nevertheless, it is important to understand the stability of such a snapshot. For that, we calculated the average absolute relative percentage change for two consecutive data collections C_1 and C_2 as follows:

$$Variability = \frac{1}{|\text{Locations}|} \sum_{l}^{\text{Locations}} \frac{|C_{1l} - C_{2l}|}{min(C_{1l}, C_{2l})}, \tag{1}$$

with C_{xl} representing the number of migrants or refugees from Venezuela living in location l at the time of collection x according to the Facebook Marketing API. Fig B shows the temporal variability of our data from May 2018 to February 2019. We also noticed a bi-weekly pattern, with the larger peaks at or close to the indicated vertical lines (every 14 days). Note that our collections were not always started at the same time of the day and sometimes exceeded 12 hours to run. Hence we believe that the actual alignment with these vertical lines is even higher, indicating a 2-week Facebook-internal refresh cycle, potentially together with smaller daily updates.

Although beyond this paper scope, Fig C shows a potential way to provide smoothed estimates avoiding sudden changes due to Facebook refresh updates. Additionally to using single-point estimates as done in Fig 3, in future work, we propose a running average that makes use of past estimates. In Fig C, we compare the single-point estimates to a running average over estimates collected in the prior 28 days. The biggest gap between these two estimates was found in late August 2018, the period in which fewer data collections were performed.

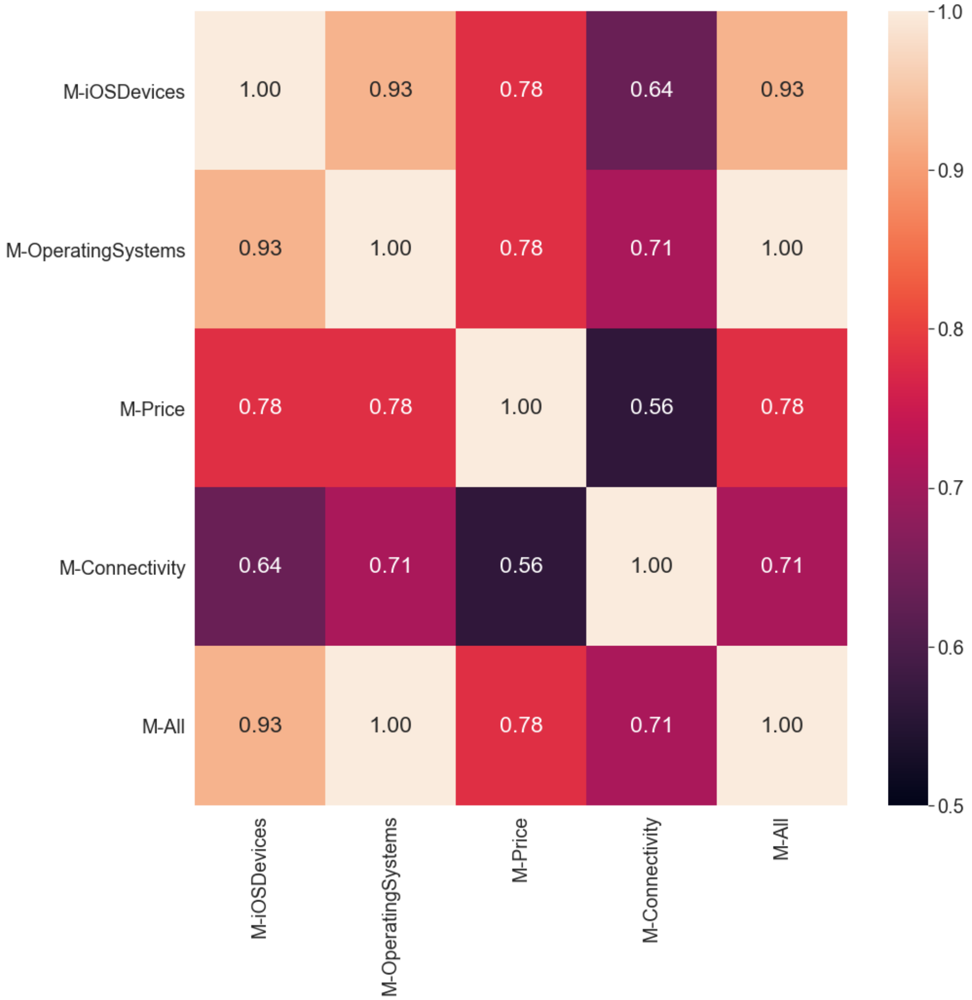

Figure A. Kendall's-τ correlation across 11 countries for the GDP estimation of the Venezuelans Migrants and Refugees among the different linear regression models. M-iOSDevices is the model trained using only the percentage of iOS devices among the target population; M-OperatingSystems, apart from the percentage of iOS devices, uses complementary percentage of Android and other devices (iOS, Android, Other); M-Price is a model created with the device category as described in Table E (Expensive, Mid-range, Cheap, Other); M-Connectivity is a model exploring the network connectivity used to access Facebook (3G, 4G, Wifi); M-All uses all variables above.

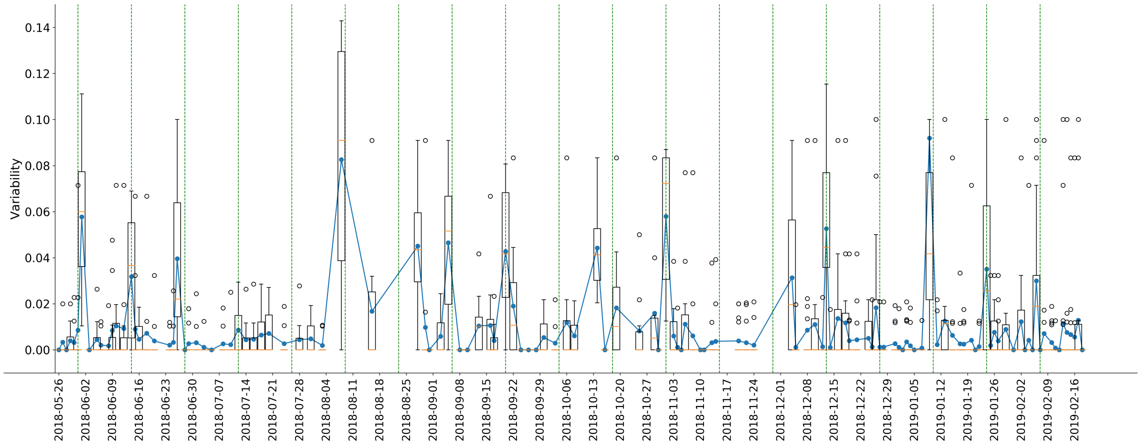

Figure B. The variability between consecutive Facebook collection over time. Each marker/boxplot represents how much variability was found between one collection and the preceding collection. We found a bi-weekly pattern, represented by the equally spaced vertical green lines, indicating when Facebook potentially refreshes their system.

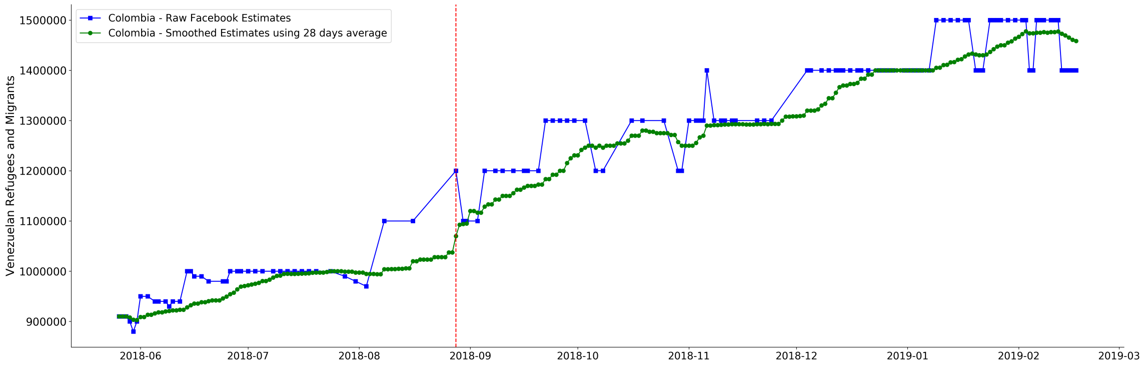

Figure C. Comparison between raw single-point Facebook estimates and smoothed estimates for Venezuelans refugees and migrants in Colombia. In the 268-day period of our study, 131 data collections were conducted. We show the raw single-point Facebook estimates (blue) and compare them to a smoothed version that averages over all collections made in the prior 28 days (green). The biggest gap between the raw estimates and the smoothed ones is shown by the red dashed vertical line.

Table A. Comparison between the estimates of refugees and migrants from Venezuela in different countries used in Fig. 1 for Facebook and both the Refugee Response Plan (RRP) and the R4V Map and Geodata (R4V).

Data Sources	Location	Facebook	Other (RRP or R4V)	Difference (FB - Other)	Rel. Diff. (FB/Other)
	Colombia	1.1M	1.3M	-163.4k	.870
Facebook data	Peru	420.0k	506.2k	-86.2k	.829
collected on	Chile	240.0k	-	-	-
Sep 3, 2018	Ecuador	180.0k	221.0k	-41.0k	.814
	Argentina	120.0k	-	-	-
RRP estimates	Panama	80.0k	-	-	-
made in	Brazil	60.0k	85.0k	-25.0k	.705
Sep 2018	Uruguay	9.1k	-	-	-
	Latin America	2.3M	2.7M	-346.2k	.870
	Colombia	1.3M	1.0M	300.0k	1.300
Facebook data	Peru	520.0k	506.0k	14.0k	1.028
collected on	Chile	260.0k	108.0k	152.0k	2.407
Nov 3, 2018	Ecuador	220.0k	221.0k	-1.0k	.995
	Argentina	130.0k	130.0k	0.0	1.000
R4V estimates	Panama	81.0k	94.0k	-13.0k	.861
made in	Brazil	73.0k	85.0k	-12.0k	.858
Nov 2018	Uruguay	9.9k	8.5k	1.4k	1.165
	Latin America	2.8M	2.4M	395.2k	1.165
	Colombia	1.4M	1.5M	-129.0k	.915
Facebook data	Peru	560.0k	698.3k	-138.3k	.801
collected on	Chile	280.0k	-	-	-
Dec 3, 2018	Ecuador	230.0k	278.0k	-48.0k	.827
	Argentina	130.0k	-	-	-
RRP estimates	Panama	80.0k	-	-	-
made in	Brazil	75.0k	103.8k	-28.8k	.722
Dec 2018	Uruguay	10.0k	-	-	-
	Latin America	3.0M	3.3M	-347.3k	.895
	Colombia	1.5M	1.1M	400.0k	1.364
Facebook data	Peru	630.0k	506.0k	124.0k	1.245
collected on	Chile	310.0k	288.0k	22.0k	1.076
Feb 2 2019	Ecuador	250.0k	221.0k	29.0k	1.131
	Argentina	140.0k	130.0k	10.0k	1.077
R4V estimates	Panama	80.0k	94.0k	-14.0k	.851
made in	Brazil	83.0k	96.0k	-13.0k	.864
Feb 2019	Uruguay	11.0k	8.5k	2.5k	1.294
	Latin America	3.2M	2.7M	513.4k	1.190

Table B. University graduate Facebook users. "University Graduate FB Users in host location" and "University Graduate FB Users from Venezuela" are the number of self-declared university graduate Facebook users from, respectively, the host population in a location and the migrants from Venezuela living in the same location. "% University Graduate Users in host location" and "% University Graduate Users from Venezuelans" are the percentage of self-declared university graduate Facebook users from, respectively, the host population in a location and the migrants from Venezuela living in the same location.

Location	University Graduate FB Users in host location	University Graduate FB Users from Venezuela	% University Graduate Users in host location	% University Graduate Users from Venezuelans
Argentina	9.1M	77.0k	30.7	54.6
Aruba	14.0k	3.3k	27.2	36.7
Bolivia	1.6M	3.5k	24.6	44.9
Brazil	35.0M	31.0k	27.6	37.8
Chile	3.5M	170.0k	29.9	54.1
Colombia	10.0M	530.0k	33.0	36.6
Costa Rica	850.0k	8.2k	27.7	49.4
Curacao	19.0k	3.4k	24.1	40.0
Dominican Republic	1.7M	25.0k	31.8	48.1
Ecuador	3.8M	110.0k	34.2	44.0
Spain	6.1M	130.0k	32.1	50.2
Guyana	91.0k	1.8k	24.3	31.6
Mexico	23.0M	47.0k	27.7	45.6
Panama	490.0k	38.0k	29.9	48.7
Peru	7.0M	290.0k	30.8	45.3
Trinidad and Tobago	250.0k	5.0k	37.9	40.7
United States	65.0M	210.0k	39.6	50.8
Uruguay	800.0k	6.0k	33.6	53.6
Venezuela	4.2M	-	35.9	-
Roraima, Brazil	82.0k	13.0k	28.3	34.9
São Paulo, Brazil	9.9M	4.0k	30.6	47.6
Miraflores, Peru	180.0k	9.6k	51.7	52.5

Table C. Details of the linear model to estimate GDP based on iOS devices (M-iOSDevices).

**Metric**	**Value**
Model	OLS
Method	Least Squares
No. Observations	15
Df Residuals	13
Df Model	1
R-squared	0.883
Adj. R-squared	0.874
F-statistic	97.76
Prob (F-statistic)	2.05e-07
Log-Likelihood	-147.59
AIC	299.2
BIC	300.6
Omnibus	0.615
Prob(Omnibus)	0.735
Skew	0.013
Kurtosis:	2.034
Durbin-Watson	1.961
Jarque-Bera (JB):	0.583
Prob(JB)	0.747
Cond. No.	8.65

Table D. Data used in the experiments to predict GDP per capita. "UN GDP'17" is the ground-truth estimations used by the linear model. "%iOS host population" and "%iOS Venezuelans" are the percentage of users that accessed Facebook with an iOS device from, respectively, the host population in a location and the refugees and migrants from Venezuela living in the same location. "GDP per capita predicted to host population" and "GDP per capita predicted to Venezelans" are the model GDP per capita predictions for both host population and Venezuelans in a location using the M-iOSDevices model.

Location	UN GDP'17	%iOS host population	GDP per capita predicted to host population	%iOS Venezuelans	GDP per capita predicted to Venezuelans
Argentina	14.4k	5.9	6.7k	15.3	16.6k
Aruba	25.7k	31.5	33.5k	18.8	20.2k
Brazil	9.8k	9.8	10.8k	4.6	5.4k
Chile	15.3k	14.6	15.9k	13.8	15.0k
Colombia	6.3k	6.8	7.6k	3.1	3.7k
Curacao	19.6k	22.6	24.2k	16.7	18.0k
Dominican Republic	7.1k	12.7	13.8k	17.5	18.9k
Ecuador	6.3k	7.3	8.2k	6.2	7.0k
Spain	28.4k	22.2	23.8k	28.5	30.4k
Mexico	9.0k	11.6	12.7k	23.2	24.9k
Panama	15.1k	8.3	9.2k	21.2	22.7k
Peru	6.6k	4.3	5.0k	4.2	5.0k
Trinidad and Tobago	16.1k	15.5	16.8k	10.8	11.8k
United States	60.1k	51.4	54.4k	54.3	57.5k
Uruguay	17.1k	13.0	14.2k	15.8	17.1k
Venezuela	-	4.4	5.1k	-	-
São Paulo, Brazil	-	12.6	13.7k	14.8	16.0k
Roraima, Brazil	-	7.5	8.4k	2.7	3.3k
Miraflores, Peru	-	16.7	18.0k	9.9	10.9k

Table E. Information regarding the mobile phone model used to access Facebook can also be used to target advertisements. We manually crafted a list of the most popular devices in December 2018 and categorize them according to their average price found in the US Amazon in that period. If a device is not found in the list it is assigned to an "Other" category. Note that a list like this requires constant maintenance to keep it updated. Facebook ID is the unique Facebook Graph API ID of each device.

Category	Device Name	Brand	Global Number of FB Users	Facebook ID	Average Top 3 Prices
Expensive	iPhone X	Apple	20,540,474	6092512462983	$991.33
	iPhone 8	Apple	16,906,896	6092512412783	$808.96
	iPhone 8 Plus	Apple	18,402,743	6092512424583	$769.52
	Galaxy Note 8	Samsung	8,164,808	6083036245383	$735.12
	Iphone 7 Plus	Apple	31,111,816	6060616598183	$561.92
	Galaxy S8+	Samsung	10,743,780	6075237226583	$544.31
	Iphone 7	Apple	46,834,282	6060616578383	$531.99
	Galaxy S8	Samsung	16,135,001	6075237200983	$511.66
Mid-Range	Galaxy S7 Edge	Samsung	16,547,729	6043522870783	$408.83
	Iphone 6S Plus	Apple	16,578,673	6031259590183	$344.99
	Ipad Mini 2	Apple	5,913,214	6011244510983	$300.31
	IPad Air 2	Apple	8,951,253	6018995113183	$289.61
	Galaxy S7	Samsung	17,334,389	6043523344783	$282.73
	Iphone 6 plus	Apple	13,859,355	6017831560783	$240.61
	Galaxy Note 3	Samsung	6,087,733	6013279353983	$236.62
	Galaxy Note 5	Samsung	6,759,041	6042330550783	$234.64
	Galaxy Note 4	Samsung	4,642,541	6019098214783	$232.74
	Ipad Air	Apple	8,433,554	6011244513583	$232.51
	Galaxy S6	Samsung	20,689,461	6026660740983	$218.98
	Ipad 3	Apple	3,770,684	6004383806772	$204.62
	Iphone 6 S	Apple	42,229,679	6031259562783	$200.91
Cheap	Iphone SE	Apple	15,263,291	6054947014783	$179.99
	Galaxy Tab 4	Samsung	5,951,716	6016925404783	$179.80
	HTC One	HTC	3,749,715	6014809859183	$179.00
	Galaxy Tab 3	Samsung	10,889,493	6016925643983	$169.00
	Ipad Mini 1	Apple	7,028,853	6011191259183	$159.00
	Iphone 6	Apple	49,648,867	6017831572183	$156.33
	Galaxy S III Mini	Samsung	3,666,046	6013017211983	$155.31
	Galaxy S5	Samsung	14,520,127	6014808618583	$154.50
	iPad 4	Apple	6,035,561	6011191254383	$152.31
	iPad 2	Apple	5,478,260	6004383808772	$139.98
	iPhone 5S	Apple	27,684,686	6010095777183	$127.05
	iPhone 5	Apple	9,745,370	6004883585572	$125.64
	Galaxy S III devices	Samsung	6,272,092	6007481031783	$116.33
	iPhone 5C	Apple	3,927,621	6010095794383	$109.83
	Galaxy S4	Samsung	9,042,092	6013016790183	$81.17
	Ipad 1	Apple	2,686,644	6004383767972	$71.80
	Galaxy Tab 2	Samsung	2,485,025	6016925657183	$40.20
	iPhone 4S	Apple	5,253,565	6004386303972	$39.15
